# Mitochondria and Aging: Redox Balance Modulation as a New Approach to the Development of Innovative Geroprotectors (Fundamental and Applied Aspects)

**DOI:** 10.3390/ijms27020842

**Published:** 2026-01-14

**Authors:** Ekaterina Mironova, Igor Kvetnoy, Sofya Balazovskaia, Viktor Antonov, Stanislav Poyarkov, Gianluigi Mazzoccoli

**Affiliations:** 1 Medical Institute, Saint-Petersburg State University, 199034 Saint-Petersburg, Russia; 2 Department of Biogerontology, Saint-Petersburg Institute of Bioregulation and Gerontology, 197110 Saint-Petersburg, Russia; 3 Department of Biochemistry, Saint-Petersburg State Pediatric Medical University, 194100 Saint-Petersburg, Russia; 4 Koltzov Institute of Developmental Biology, Russian Academy of Sciences, 119334 Moscow, Russia; 5 Foundation Istituto di Ricovero e Cura a Carattere Scientifico Casa Sollievo della Sofferenza, Opera di Padre Pio da Pietrelcina, 71013 San Giovanni Rotondo, Italy

**Keywords:** mitochondria, redox regulation, oxidative stress, antioxidants, glutathione, aging

## Abstract

Redox (reduction–oxidation) processes underlie all forms of life and are a universal regulatory mechanism that maintains homeostasis and adapts the organism to changes in the internal and external environments. From capturing solar energy in photosynthesis and oxygen generation to fine-tuning cellular metabolism, redox reactions are key determinants of life activity. Proteins containing sulfur- and selenium-containing amino acid residues play a crucial role in redox regulation. Their reversible oxidation by physiological oxidants, such as hydrogen peroxide (H_2_O_2_), plays the role of molecular switches that control enzymatic activity, protein structure, and signaling cascades. This enables rapid and flexible cellular responses to a wide range of stimuli—from growth factors and nutrient signals to toxins and stressors. Mitochondria, the main energy organelles and also the major sources of reactive oxygen species (ROS), play a special role in redox balance. On the one hand, mitochondrial ROS function as signaling molecules, regulating cellular processes, including proliferation, apoptosis, and immune response, while, on the other hand, their excessive accumulation leads to oxidative stress, damage to biomolecules, and the development of pathological processes. So, mitochondria act not only as a “generator” of redox signals but also as a central link in maintaining cellular and systemic redox homeostasis. Redox signaling forms a multi-layered cybernetic system, which includes signal perception, activation of signaling pathways, the initiation of physiological responses, and feedback regulatory mechanisms. At the molecular level, this is manifested by changes in the activity of redox-regulated proteins of which the redox proteome consists, thereby affecting the epigenetic landscape and gene expression. Physiological processes at all levels of biological organization—from subcellular to systemic—are controlled by redox mechanisms. Studying these processes opens a way to understanding the universal principles of life activity and identifying the biochemical mechanisms whose disruption causes the occurrence and development of pathological reactions. It is important to emphasize that new approaches to redox balance modulation are now actively developed, ranging from antioxidant therapy and targeted intervention on mitochondria to pharmacological and nutraceutical regulation of signaling pathways. This article analyzes the pivotal role of redox balance and its regulation at various levels of living organisms—from molecular and cellular to tissue, organ, and organismal levels—with a special emphasis on the role of mitochondria and modern strategies for influencing redox homeostasis.

## 1. Introduction

Modern theories of aging are based on a close connection of this process with disruptions in the interaction of a living organism’s genome with the exposome—a complex set of environmental exposures, including physical, chemical, and biological factors.

The evolutionarily shaped systems of interaction between the exposome and the living organism underlie the organism’s adaptation to changing environmental conditions.

With age, the body’s adaptive responses decline, which leads to a disruption in its regenerative and protective capabilities, resulting in the development of age-associated diseases and a deterioration in overall health [1].

**Figure 1:**
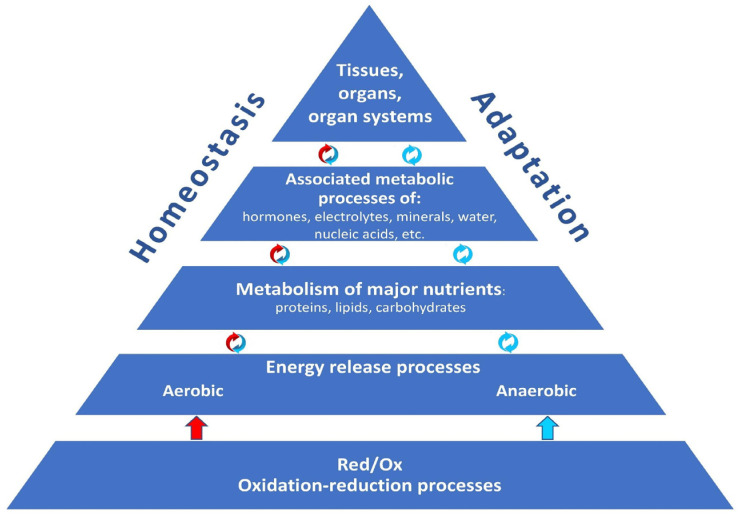
The fundamental role of redox reactions in maintaining the body’s adaptive capacities. Oxidation-reduction reactions (Red/Ox) form the basis for the release of energy required for the functioning of biological systems at all levels of organization, including the metabolism of essential nutrients (proteins, lipids, carbohydrates), which determine the structure and functioning of sub-cellular structures, cells, and supracellular structures (organs, organ systems, and the organism as a whole). Aerobic energy release (color-coded red) is combined with anaerobic processes (color-coded blue) to support the body’s vital functions. Anaerobic energy release is capable of ensuring the full ac-tivity of a biological system.

The body’s adaptive processes are inextricably intertwined with oxidation–reduction (redox) reactions, as they ensure the release of energy for life activity and homeostasis maintenance. Actually, redox reactions, with their sufficient and necessary activity, are the foundation upon which the whole body’s structural and metabolic organization, homeostasis, and adaptive capacities are based (Figure 1).

Mitochondria play a key role in maintaining homeostasis and in the processes of the organism’s adaptation to external and internal environmental changes, releasing energy through the coupling of redox reactions with the formation of ATP (adenosine triphosphate). Adaptive changes in the mitochondria themselves help the body respond to stress, hypoxia, and other negative impacts, influencing metabolism, defense mechanisms, and behavioral responses.

However, mitochondria are the main sources and targets of ROS, which predetermines their important role in the aging process. Impaired mitochondrial quality and dynamics (fission and fusion processes), as well as a decrease in the activity of antioxidant systems such as the thioredoxin system and the NADPH-dependent system, lead to accumulation of damage that accelerates cellular and tissue aging.

Oxidation–reduction (redox) reactions are an integral part of balanced metabolism and physiological processes, including cell proliferation, differentiation, and death. The physicochemical basis of the redox reaction is electron transfer (a chemical entity capable of donating electrons is a reducing agent, and a chemical entity capable of accepting electrons is an oxidizing agent).

Disruption of redox status and antioxidant defense greatly contributes to the development of age-associated diseases such as diabetes, cardiovascular diseases, and neurodegenerative disorders, and also leads to decreased cell viability. These factors point to the importance of maintaining mitochondrial functions and, above all, redox homeostasis for a potential slowing of the aging process [2].

The basis of the cellular aging process is a disruption of mitochondrial quality control, including the mechanisms responsible for the removal of damaged mitochondria through mitophagy. A decreased mitophagy efficiency leads to the accumulation of dysfunctional mitochondria, thereby accelerating cellular aging.

Oxidative stress and redox imbalance result in damage to DNA, proteins, and lipids, which affects the aging phenotype, including loss of cellular function and a higher likelihood of triggering cell death.

In this regard, it is necessary to emphasize the importance of redox modulation as a promising approach to the development of innovative geroprotectors—substances that can contribute to extending lifespan.

## 2. The Role of Mitochondria and Redox Status in Cellular Function

Mitochondria play a central role in cellular energy metabolism. They provide approximately 90% of ATP production through oxidative phosphorylation. However, this energy production mechanism is accompanied by electron leakage from the respiratory chain, which leads to the formation of ROS as byproducts—superoxide anion (O_2_•^−^), hydrogen peroxide (H_2_O_2_), and hydroxyl radical (•OH) [3]. The balance between ROS production and the ability of antioxidant systems to neutralize them determines the redox status of the cell.

When ROS levels exceed a cell’s antioxidant capacity, oxidative stress occurs, which can lead to various consequences, including damage to cellular structures and serious impairment of their functions [4].

At low physiological concentrations, ROS act as second messengers and perform important functions in redox signaling, regulating key cellular processes—proliferation, differentiation, apoptosis, and adaptation to stress [5]. Thus, hydrogen peroxide and superoxide anion are important factors involved in the control of insulin synthesis and secretion in pancreatic β-cells.

Conversely, excess ROSs damage cellular molecules: proteins (oxidation of cysteine residues), lipids (peroxidation), nucleic acids (DNA damage), and other macromolecules, as well as organelles and cells as a whole, leading to their dysfunction. For example, the hydroxyl radical is known as a particularly reactive oxidant, capable of destroying phospholipids in cell membranes and proteins. Such damage can initiate signaling cascades that lead to the activation of proliferative and apoptotic pathways, which contributes to disruption of cellular homeostasis, the development of neurodegenerative disorders, cardiovascular diseases, tumors, and cell death [6].

At the molecular level, redox status is determined by the balance between oxidizing and reducing agents, such as NAD^+^/NADH and GSH/GSSG, reduced and oxidized glutathione, the ratio of which serves as an important marker of the redox status of the cell [7].

Antioxidants such as glutathione, ascorbic acid, and tocopherols act as protective agents, neutralizing excessive ROS and preventing damage to cell components.

Glutathione, for instance, exists in two forms: reduced (GSH) and oxidized (GSSG), and the ratio of these forms serves as an important marker of cell redox status.

The normal functioning of antioxidant systems depends on the presence of ROS molecules such as NADPH, which provides reducing equivalents for the synthesis of GSH and other antioxidants. The signaling role of ROS has been confirmed by numerous studies using genetically encoded redox sensors, pharmacological inhibitors, and knockout models of antioxidant enzyme genes. However, most data on their damaging role were obtained in vitro using high, non-physiological doses of prooxidants, requiring cautious extrapolation to in vivo models of aging.

The main sites of ROS production are complexes I and III of the mitochondrial respiratory chain, and the intensity of their formation depends on the redox state of the NADH and ubiquinone pools, the transmembrane potential (Δψm), and oxygen availability. To protect against their own ROS, mitochondria have an autonomous antioxidant system that includes matrix Mn-dependent superoxide dismutase (SOD2), which converts O_2_•^−^ into H_2_O_2_; peroxiredoxins (Prx3, Prx5) and glutaredoxins, which detoxify H_2_O_2_ and organic peroxides with the help of GSH; glutathione peroxidase 1 (GPx1) and catalase. These enzymes play a key role in neutralizing ROS, preventing their accumulation and thereby protecting cellular structures from oxidative damage, and the efficiency of the entire system critically depends on the constant influx of reducing equivalents (e.g., NADPH) from the cytosol [6].

Mitochondria interact with peroxisomes, which are involved in lipid metabolism and hydrogen peroxide detoxification. Together, they form a complex network of redox signals that allows cells to adapt to changes in the microenvironment and maintain redox homeostasis. Activation of redox signaling can lead to increased production of transcription factors, such as NRF2, which regulate the expression of genes responsible for antioxidant synthesis and other protective mechanisms. For example, under conditions of oxidative stress, peroxisomes can enhance hydrogen peroxide degradation, thereby reducing ROS levels, which helps protect mitochondria and other cellular structures from damage [8].

The effectiveness of mitochondrial antioxidant systems is critical for maintaining cellular function and preventing pathological conditions. Redox imbalances occurring through changes in mitochondrial membrane permeability and overexpression of various caspases can lead to the activation of cell death pathways that trigger apoptosis or necrosis.

However, although studies suggest that enhanced antioxidant defenses should improve and prolong viability, genetic manipulations to overexpress antioxidant enzymes, such as SOD2, in model organisms yield mixed results. These results highlight the idea that simply reducing systemic ROS levels can disrupt their important signaling functions required for the healthy functioning of vital processes. An important unanswered question arises: how does the cell spatially and temporally separate signaling and damaging ROS pools, and how can ROS levels be most effectively influenced without disrupting signaling? Current views are shifting toward the concept of mitochondrial hormesis: low, physiological levels of ROS are necessary for the activation of protective transcriptional programs, such as NRF2 and PGC-1α, which maintain homeostasis. The implication is that the problem of oxidative stress and subsequent impairment lies not simply in the amount of ROS, but in the loss of the capacity for redox signaling and adaptive response to stress.

Thus, extensive evidence suggests that mitochondria are key organelles involved in aging mechanisms and represent promising targets for developing new therapeutic approaches aimed at ensuring normal cellular function and slowing the aging process.

## 3. Mitochondrial Dysfunction and Inflammaging

Mitochondrial dysfunction and oxidative stress are involved in the mechanisms of aging and pathogenesis of age-associated diseases.

Mitochondrial dysfunction results in excessive ROS production, damaging mitochondrial DNA (mtDNA) and disrupting energy metabolism [9]. Such changes initiate the activation of inflammatory signaling cascades through the formation of NF-κB and NLRP3 inflammasomes, which contribute to the development of inflammaging [10]. This vicious cycle, where damage caused by oxidative stress continues to impair mitochondrial function, creates conditions for the progression of various pathologies.

These processes are closely associated with inflammaging—a chronic aseptic inflammation that triggers cellular aging through forming a specific immunophenotype, which is characterized by a molecular imbalance—predominant proinflammatory cytokine production and a lack of endogenous geroprotectors [11].

Inflammaging, as a consequence of innate immune activation via mitochondrial-derived damage-associated molecular patterns (DAMPs), becomes chronic and accelerates tissue aging. The role of inflammaging in the development of age-associated pathologies has been described in detail for neurodegenerative processes, metabolic syndrome, diabetes, autoimmune processes, sarcopenia [12,13,14], and other pathologies.

## 4. Molecular Logistics of Mitochondrial Aging: The Contribution of Membrane Proteins

Mitochondrial aging is associated with disruptions in mitochondrial structure, function, and proteostasis (the process of maintaining a sufficient protein pool for cell activity), leading to decreased energy metabolism and the development of age-associated diseases.

Mitochondrial membrane proteins, located on both the outer and inner membranes, play a central role in regulating organelle fusion and fission processes, allowing them to adapt to changes in metabolic conditions, ensuring the removal of damaged areas, functional integrity, and renewal of organelles [15]. Disruption of protein function initiates and catalyzes the aging process, leading to significant disturbances in mitochondrial dynamics. For example, dysfunction of proteins responsible for fusion can lead to the formation of long, undivided mitochondria (the effect of anomaly of the DRP1 protein—Dynamin-related protein 1), which complicates their renewal and the removal of damaged areas. At the same time, disturbances in proteins such as MFN1 and MFN2 (Mitofusins 1 and 2) can hinder the restoration of mitochondrial functionality and contribute to their degradation. The accumulation of damaged mitochondria caused by these disorders leads to a decrease in membrane potential and an increase in ROS production [16]. In turn, excessive ROS formation causes oxidative stress, which aggravates mitochondrial dysfunction and initiates cascades of cellular damage that contribute to the development of diseases associated with aging.

Certain membrane proteins, such as SAMM50 (Sorting and Assembly Machinery protein 50) and ATAD3 (ATPase family AAA domain-containing protein 3), are involved in the endosomal-autophagic pathway, which ensures the degradation of damaged mitochondria and prevents the accumulation of mutations, maintaining mitochondrial functional integrity and cell viability [17].

**Figure 2:**
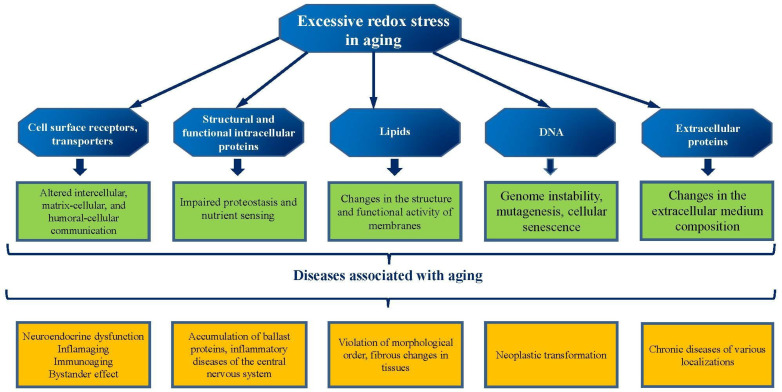
Molecular targets, negative redox modulation is associated with typical healthy manifestations of aging.

As mentioned above, the effectiveness of mitochondrial quality control mechanisms is directly linked to proteostasis—the system that ensures proper protein folding, assembly, and degradation. Thus, maintaining proteostasis and the functional activity of membrane proteins is a crucial factor in preventing mitochondrial dysfunction and associated diseases (Figure 2).

The mitochondrial quality control system is responsible for the regulation and operation of a complex mechanism that ensures the selective removal of damaged or misfolded proteins. This supports the functional integrity of organelles. Mitochondrial-derived compartments (MDCs) and mitophagy play a key role in this process. MDCs function as specialized structures that are involved in the degradation of membrane proteins, ensuring their sorting and removal from the mitochondria.

This process also prevents the accumulation of damaged proteins in cells. The efficiency of mitophagy depends on various signaling pathways, including the activation of proteins such as PINK1 and Parkin, which are responsible for identifying and marking damaged mitochondria for subsequent removal. Disruptions in proteostasis and mitophagy are associated with accelerated aging and the development of neurodegenerative and metabolic diseases, which points to the need to maintain their functional activity to prevent cellular dysfunction [18].

With age, structural and functional changes are observed in the protein complexes of the respiratory chain and ATP synthase, resulting in a reduced efficiency of energy metabolism. These changes can be caused by both the accumulation of damaged proteins and disruptions in mitochondrial fission and fusion, which impede mitochondrial population renewal and increase the likelihood of oxidative stress.

Dysfunction of the protein complexes of the respiratory chain leads to decreased ATP synthesis, which has a negative impact on cellular energy metabolism and contributes to the development of various pathologies, including age-associated neurodegenerative diseases [19].

Mitochondria-associated membranes (MAMs), which are formed between mitochondria and the endoplasmic reticulum (ER), play an important role in regulating numerous cellular processes, including lipid metabolism, calcium homeostasis, and autophagy. These structures mediate the interaction between mitochondria and the ER, enabling the coordination of metabolism and signals between the two organelles [20].

Dysfunction of the MAM can lead to disruption of these processes, which, in turn, contributes to cellular aging and the development of associated diseases, such as metabolic syndrome and neurodegenerative disorders. Maintaining MAM stability is a dynamically developing field, but the sequence and hierarchy of these disruptions remain unclear. There is debate as to whether primary age-associated MAM dysfunction disrupts calcium and lipid homeostasis, which secondarily leads to mitochondrial dysfunction, or whether, conversely, the age-related decline in mitochondrial membrane potential disrupts calcium reuptake into the ER, destabilizing the MAM. Current evidence suggests that the MAM is not simply a contact site, but a critical signaling platform where metabolic signals are integrated. Disruption of this integration may be one of the earliest events in cellular aging, preceding mitochondrial failure, but the molecular details require further study.

However, the connection is known, so maintaining the functional activity of membrane proteins, effective quality control and dynamics of mitochondria, as well as the integrity of mitochondria-associated membranes, is critical for ensuring cellular homeostasis and slowing down the aging process [21].

## 5. Molecular Geroprotectors: Modern Approaches to Slowing Senescence

Geroprotectors are substances that slow the aging process, improve quality of life, and reduce the risk of developing age-associated diseases. Furthermore, geroprotectors should stimulate the synthesis of signaling molecules whose expression decreases with age [22].

The development and widespread implementation of molecular and cellular technologies in biomedicine have helped identify a number of potential compounds that could act as geroprotectors [23].

Among these, flavonoids are the most common class of substances demonstrating geroprotective activity. Thus, the administration of flavonoid C- and O-glycosides isolated from an aerial part of *L. hedysaroides* into a culture of HuTu80 cancer cells resulted in the inhibition of the α-N-acetylgalactosaminidase enzyme which is a recognized therapeutic target in oncology [24].

Another promising flavonoid, dihydroquercetin, isolated from coniferous plants, is a potential herbal remedy for preventing the aging processes. A study of the effects of a modified form of this substance on the central nervous system (CNS) of animals of different ages, for neuroinflammation modeling, demonstrated a therapeutic effect of dihydroquercetin [25].

The administration of dihydroquercetin contributed to the improvement of morphological parameters of blood vessels in the substantia nigra of the brain, including their total length, area, and number of bifurcations. Furthermore, a pronounced effect was observed in older animals under conditions of intense neuroinflammation. A study assessing the effectiveness of polyphenols on the rate of H_2_O_2_ generation in rats with liver injury showed that dihydroquercetin, pinosylvin, and dihydromyricetin reduced mitochondrial ROS production, demonstrating the antioxidant effect of these substances [26].

Another well-known polyphenol, resveratrol, also has geroprotective potential. The main beneficial effects of this substance include cardioprotective, vasodilatory, and antidiabetic actions [27]. Studies of resveratrol modifications have shown their cytoprotective effect, mediated through various molecular mechanisms. Thus, monomethylresveratrol reduced the caspase-3 activity and effectively removed superoxide through mitophagy. Oxyresveratrol also induced cytoprotection, but without mitophagy activation [27].

A randomized clinical trial of the effects of short-term resveratrol treatment of patients with hypertension showed increased levels of nitric oxide, a key molecule in vasodilation, but this did not lead to blood pressure reduction [28].

Regretfully, all of the above-mentioned flavonoids have limited clinical application due to a number of factors, including low solubility and bioavailability, the need for purification and labor-intensive extraction from plants, as well as the lack of epidemiological studies of this group of substances [29].

A major problem with flavonoids is their poor pharmacokinetics, and therefore the biosynthesis of modified flavonoids with more favorable biophysical and pharmacokinetic properties for clinical application is an important task in modern geriatrics [30].

Of course, one of the previously mentioned processes involved in the development of pathological aging is inflammaging. The NF-κB signaling pathway is a key link in the interplay of inflammation and aging processes—its hyperactivation leads to the production of proinflammatory cytokines and the development of a chronic inflammatory phenotype. The use of substances that inhibit this pathway, while maintaining a balance between reducing inflammation and preserving physiological immune responses, is a promising option for fighting pathological aging.

Melatonin is a geroprotector and a circadian rhythm regulator, exhibiting powerful antioxidant, immunomodulatory, and anti-inflammatory activity. A study of melatonin effects on the NF-κB signaling pathway revealed its limited impact, yet melatonin is capable of inhibiting biomolecules controlled by this pathway.

The combined use of melatonin and anticancer drugs such as temozolomide and cisplatin significantly reduced tumor cell proliferation, migration, and invasion [31], and also helped reduce the toxic effects of cytostatic drugs and tissue destruction by inhibiting COX-2 production [32]. Melatonin has also been shown to stimulate lifespan in *Drosophila melanogaster*, with the greatest effect observed in mutant lines (*Sod* and *mus210*), which confirms the ability of this geroprotector to detoxify free radicals and decrease oxidative stress [33].

Bioavailable geroprotectors are represented by a group of low molecular weight antioxidants—vitamins A, E, and C. Retinol, a vitamin A derivative, is believed to be one of the most effective substances for fighting against skin aging. Retinol can stimulate collagen synthesis, inhibit matrix metalloproteinase (MMPs) activity, reduce oxidative stress, and modulate the expression of skin renewal genes [34].

Research into the effects of these vitamins on T-cell proliferation, oxidative status, and cytokine production in older adults demonstrated that administration of vitamins C and E significantly improved these parameters, reducing intracellular oxidative stress which is observed in elderly people due to depletion of the GSH system [35].

Furthermore, vitamin C stimulates the activity of several transcription factors (Nrf2, Ref-1, AP-1), which promote the expression of genes encoding antioxidant proteins. Another function is to support the action of other exogenous antioxidants, primarily polyphenols [36].

Rapamycin, an immunosuppressant and mTOR inhibitor, is a key regulator of cellular growth, metabolism, and aging. Rapamycin binds to its receptor, the cytoplasmic protein FKBP12 (FK-binding protein 12), after which this complex verifies the FRB domain (FKBP12-Rapamycin binding domain) of mTOR complex 1 (mTORC1), leading to destabilization of the complex.

Moreover, rapamycin is capable of suppressing the response to IL-2, preventing the activation of T and B cells. The inhibition of mTOR complex 1 (mTORC1) signaling increases lifespan in yeast, worms, flies, and mice [37]. Rapamycin also improves the condition of mice in an inflammaging model, with positive effects including improved long-term memory, better neuromuscular coordination, and restoration of tissue architecture [38]. The use of rapamycin as a geroprotector is still at the stage of clinical trials, while researchers are looking for the optimal dosage regimen and its analogs with milder side effects.

So, despite the impressive geroprotective potential demonstrated by compounds from the flavonoid group, mTOR inhibitors (rapamycin), and circadian rhythm regulators (melatonin), their widespread clinical use faces such challenges as low bioavailability, side effects, and the lack of large-scale randomized trials in humans.

## 6. Retinol as a Well-Known Geroprotector for the Skin

Retinol, a vitamin A derivative, is considered one of the most effective anti-aging agents and one of the most studied geroprotectors with a proven effect on the rate and phenotype of cellular aging. Traditionally, the geroprotective effects of retinol and its active metabolite, all-trans-retinoic acid (ATRA), have been associated with their ability to activate the nuclear receptors RAR and RXR. This complex, by binding to specific DNA sequences (RARE), modulates the expression of hundreds of genes involved in differentiation, proliferation, and apoptosis. Retinol can also stimulate collagen synthesis, inhibit the activity of MMPs, reduce oxidative stress, maintain genomic integrity, and regulate the balance of synthesis and degradation of the extracellular matrix (ECM) [34]. The main challenges in the use of retinoids remain their photoinstability, skin irritation, and systemic toxicity at high doses. Current experimental research is focused on developing precise solutions to these problems, confirming their key role as targets for intervention. A key advance is the development of selective RAR-γ agonists, such as trifarotene. Since RAR-γ is the predominant receptor subtype in the epidermis, its selective activation enables targeted enhancement of keratinocyte proliferation and differentiation, extracellular matrix remodeling, and suppression of inflammation, while minimizing the side effects associated with activation of other receptor subtypes (RAR-α/β) [39].

An important experimental discovery was the influence of retinoids on the skin and intestinal microbiome, which, in turn, modulates the local and systemic inflammatory environment (inflammation) https://pmc.ncbi.nlm.nih.gov/articles/PMC12114363/ (accessed on 5 October 2025). This highlights the role of retinol not only as a direct agent but also as a modulator of the body’s ecosystem that contributes to aging. It was also found that ATRA is capable of inducing DNA demethylation and post-translational histone modifications, such as acetylation, exerting a long-term effect on the expression patterns of aging-related genes, such as p16 and p21, without altering the nucleotide sequence itself. Not to be overlooked is the bidirectional relationship between the retinoid pathway and key kinases responsible for the cellular response to stress and aging, including the MAPK, Akt, and AMPK pathways. These interactions allow retinoids to rapidly adapt cellular responses under conditions of oxidative or metabolic stress, acting as an integrating signal [39].

Thus, retinol and its derivatives act not as single-target agents, but as multiplex modulators capable of simultaneously influencing several interconnected mechanisms of aging.

## 7. Vitamin C as a Geroprotector

Among exogenous antioxidants, vitamin C (ascorbic acid) stands out not only for its fundamental biochemical role but also for its proven ability to correct specific signs of skin aging, particularly photoaging. Unlike retinoids, which act primarily through nuclear receptors, vitamin C realizes its geroprotective potential through direct antioxidant action and enzymatic activity, making it a versatile modulator of the extracellular matrix and cellular response to stress. Vitamin C’s geroprotective effects are based on its unique chemical properties and its essential role in biochemical processes critical for maintaining youthful skin. These mechanisms include: stimulation of collagen synthesis and stabilization, suppression of extracellular matrix degradation, direct antioxidant and brightening action, support for the regeneration of the epidermal barrier and immune response [40].

The main problem with vitamin C use in dermatology is its chemical instability, easy oxidation in air and light, and poor penetration through the stratum corneum of the epidermis in its active form. Therefore, research is focused on the development of stable formulations and delivery technologies. A 2024 clinical trial directly assessed the effect of pure ascorbic acid on signs of photoaging using methods that increase skin permeability [41]. The study involved 25 patients with sensitive and erythematous skin. Vitamin C was delivered to one side of the face using sonophoresis (2 MHz ultrasound) and to the other using microneedling (0.2 mm needles). Both methods showed statistically significant improvements: increased skin elasticity (R5 parameter on a cutometer) and reduced erythema. The greatest reduction in cheek redness was observed with the combination of microneedling. This study is critically important because it demonstrates that even on reactive skin, vitamin C delivered to the dermis at adequate concentrations is effective and safe without exacerbating irritation. Furthermore, vitamin C stimulates the activity of several transcription factors (Nrf2, Ref-1, AP-1), which ensures the expression of genes encoding antioxidant proteins. An important function is to support the action of other exogenous antioxidants, primarily polyphenols [36].

## 8. Melatonin as a Systemic Geroprotector

In the context of a systemic approach to geroprotection aimed at correcting various mechanisms of cellular aging, such as oxidative stress, mitochondrial dysfunction, impaired proteostasis, and chronic inflammation, melatonin (N-acetyl-5-methoxytryptamine) represents a unique multifaceted compound. As a central hormone of the circadian system, it is also a potent endogenous antioxidant with pronounced anti-inflammatory, immunomodulatory, and protective properties, synthesized not only in the pineal gland but also in peripheral tissues, including the skin. An age-related decline in its production directly correlates with impaired homeostasis and the progression of age-associated pathologies, making it a strategically important target for anti-aging therapy [42].

Melatonin and its metabolites are known to significantly reduce oxidative stress in aging cells or cells exposed to toxins. Oxidative damage results from the formation of free radicals in cells, particularly in the mitochondria. In recent years, it has been discovered that melatonin, a powerful antioxidant, is found in higher concentrations in mitochondria than in other organelles or subcellular locations. Recent data suggest that mitochondrial membranes possess transporters that facilitate the rapid uptake of melatonin by these organelles against the cell gradient. High melatonin levels in mitochondria are biologically optimal, as these organelles produce large quantities of free radicals. Therefore, intracellular melatonin production favorably neutralizes free radicals and reduces oxidative damage [43]. The authors believe that high melatonin levels in mitochondria will protect the body from premature aging and the development of age-related diseases.

Melatonin’s geroprotective effects are diverse and enable it to address key drivers of cellular aging. Firstly, this hormone is an effective endogenous free radical scavenger through the formation of antioxidant cascades: when neutralizing ROS, it is sequentially metabolized into AFMK and AMK compounds, which also possess high antioxidant activity, significantly enhancing the overall protective effect [44]. Furthermore, melatonin activates the expression and activity of key endogenous antioxidant enzymes, such as SOD, GPx, and CAT. Secondly, melatonin actively accumulates in mitochondria, exerting a stabilizing effect on them. It protects respiratory chains from oxidative damage, reduces electron leakage and mitochondrial ROS production, and increases the efficiency of oxidative phosphorylation and ATP synthesis. It also inhibits the mitochondrial apoptotic pathway, preventing cell death under stress. Thirdly, disruption of circadian rhythms is known to be an independent factor in accelerated aging, as it leads to desynchronization of metabolic and reparative processes.

Restoring rhythms with melatonin promotes the optimization of genomic function and may influence the epigenetic regulation of genes associated with aging [45]. Fourth, this hormone suppresses the activation of key pro-inflammatory transcription factors, such as NF-κB, and reduces the production of pro-inflammatory cytokines IL-1β, IL-6, and TNF-α. It modulates immune function, potentiating the immune response and limiting excessive inflammation, which allows it to be characterized as an “immune buffer.” This is especially important for the fight against inflammaging. Fifth, melatonin has been shown to reduce the level of DNA damage induced by ultraviolet radiation and oxidative stress, including the formation of cyclobutane pyrimidine dimers and 8-hydroxy-2′-deoxyguanosine. This effect is associated with both its direct antioxidant effect and the activation of DNA repair systems. Melatonin also inhibits excessive apoptosis in skin cells (keratinocytes and fibroblasts) and neurons, maintaining the cell pool [42].

Melatonin production declines with age, causing a deficiency that leads to sleep disturbances, weakened antioxidant defenses, accumulation of oxidative damage, and the progression of age-related diseases. A current area of research is its potential to reduce the risk of neurodegenerative and cardiovascular diseases, as well as tumor development [46]. In cosmeceuticals, melatonin is considered an active component with proven anti-aging, moisturizing, and anti-inflammatory effects.

To study the effect of long-term melatonin administration on benzopropene-induced carcinogenesis and the lifespan of experimental animal models—mice [46]. After 26 weeks of experiments in which mice were given the carcinogen, melatonin, and metformin, the data obtained demonstrated a statistically significant geroprotective effect of melatonin: an increase in the average lifespan and the latent period of tumor formation (an important indicator of geroprotection is the delay in the onset of age-related diseases), a decrease in the incidence and multiplicity of tumors (the incidence of tumors in the melatonin group was 67% versus 83% in the control).

Unlike many exogenous agents, melatonin is a natural regulator in the body, which increases the potential for its use. The most effective strategies are topical application for the prevention and correction of skin aging and systemic use to correct age-related deficiencies and related disorders.

Synthetic melatonin is available as medications and dietary supplements. Dozens of brand names are available on the pharmaceutical market, including medications with proven efficacy for certain circadian rhythm disorders, sleep-normalizing agents (hypnotics and sedatives), as well as adaptogens and general tonics—combinations containing melatonin and other active ingredients designed to increase the body’s resilience to stress and normalize neuroendocrine function.

Melatonin in these medications is a complete analog of the endogenous hormone. After oral administration, it is rapidly absorbed, and its maximum plasma concentration is reached within 20–60 min on average. Bioavailability is relatively low (approximately 15%) due to the “first-pass” effect in the liver, where it is metabolized into inactive forms. Its half-life is short—approximately 45 min—which explains its primary use for facilitating sleep onset rather than maintaining sleep throughout the night.

Its primary mechanism of action involves binding to specific melatonin receptors, MT1 and MT2, in the suprachiasmatic nucleus of the hypothalamus, the body’s primary “biological clock.” This interaction suppresses wakefulness signals emanating from this nucleus; indirectly increases GABA (the main inhibitory neurotransmitter) and serotonin concentrations in the midbrain and hypothalamus; and promotes a decrease in body temperature and the onset of drowsiness [43].

In addition to inducing sleep, melatonin preparations have adaptogenic and mild sedative properties, helping to reduce stress responses and regulate neuroendocrine functions. Melatonin can also interact with many medications, potentiating the effects of sedatives and anticoagulants, such as warfarin, and reducing the effectiveness of antihypertensive and immunosuppressive medications.

It is worth noting that although melatonin preparations demonstrate significant geroprotective potential in experimental models due to their antioxidant and circadian activity, their direct use in clinical practice for slowing aging is limited. The primary medical indication remains sleep and circadian rhythm disorders. Long-term use as a geroprotector requires further large-scale clinical trials to assess the benefit-risk balance.

## 9. Redox Status Modulation During Aging: Prevention of Accelerated Organ and Tissue Involution

A change in redox status is viewed as one of the key triggers for the development of accelerated and pathological aging, and its targeted modulation can be a strategy for slowing these processes and maintaining the functional activity of tissues at an evolutionarily determined level [47]. The most promising strategy for maintaining the balance between oxidative and reductive processes in the body during aging is the activation of the endogenous antioxidant system.

An impact on the KEAP1-NRF2 signaling pathway (a regulator of antioxidant gene transcription) activates GSH production and associated enzyme systems (glutathione-S-transferase, glutathione peroxidase, glutathione reductase), as well as SOD enzymes, catalase, and heme oxygenase-1 [48]. Thus, the targeted pharmacological activation of NRF2 in endothelial progenitor cells of aged mice protects these cells from oxidative stress, reduces their biological dysfunction, and decreases NLRP3 inflammasome expression [49].

In birds with high levels of ROS, activation of this pathway is an adaptive mechanism for combating oxidative stress [50]. In male fruit flies, mutations leading to loss of function of the KEAP1 gene have a significant positive effect on resistance to oxidative stress and lifespan [51].

A major protective antioxidant mechanism activated by the KEAP1-NRF2 system is the induction of the expression of GPX2, PRDX2, TXN1, and SRXN1 genes, whose biological role is to maintain redox homeostasis through the activation of GCLC, GSLM, and GST, which are involved in GSH biosynthesis; while NQO1 and CYP2A6 are involved in xenobiotic detoxification [52].

Another important function of this pathway is the regulation of GSH synthesis: its activation leads to the expression of the genes encoding the catalytic subunits of glutamate-cysteine ligase (GCLC) and glutathione synthetase (GSS), which are key enzymes in GSH synthesis, also resulting in increased activity of glutathione reductase (GSR), which restores oxidized glutathione.

NRF2 knockout (NRF2^−/−^) mouse offspring exhibited low intracellular and extracellular GSH levels in astrocytes as compared to glial cells cultured from NRF2 wild-type (NRF2^+/+^) offspring [53]. It is important to account for the dual role of prolonged and excessive NRF2 activation: it can promote malignant cell survival and chemotherapy resistance [54]. Therefore, approaches aimed at its temporary or cyclic activation, mimicking the body’s natural rhythms, are believed to be promising.

Another way to activate the endogenous antioxidant system is to replenish the NAD^+^ pool, an essential cofactor for many enzymatic reactions. Its level is critical for sirtuin synthesis and DNA repair. The NAD^+^ level decreases significantly with age, and this is also one of the causes of aging and the development of age-associated diseases.

Aging has been shown to be accompanied by a decrease in the NAD^+^/NADH ratio in human plasma due to the depletion of NAD^+^ stores, rather than as a consequence of an increase in NADH content [55].

Sirtuins (SIRT1–6) are NAD^+^-dependent deacetylases that catalyze the removal of the acetyl group from lysines on histones and proteins, playing a key role in regulating genome stability, energy homeostasis, and signaling mechanisms for anti-stress and geroprotective functions [56].

It has been established that individuals with low SIRT1 levels in childhood are prone to the development of premature cardiovascular dysfunction in adulthood [57]. NAD^+^ deficiency during aging leads to oxidative tissue damage, decreased metabolism, disruption of circadian rhythms, and the development of mitochondrial dysfunction due to dysregulation of sirtuin expression and disruption of signaling pathways that involve p53, NF-κB, PGC-1α, and HIF-1α [58].

The development of these pathophysiological mechanisms shortens lifespan. It has been noted that increasing intracellular NAD^+^ levels can be a therapeutic strategy for combating pathological aging. In this case, taking NAD^+^ is ineffective, since it is not absorbed by cells, so it is advisable to implement this strategy through ways of increasing the level of NAD^+^ precursors (nicotinamide mononucleotide and nicotinamide riboside) and controlling the process of biosynthesis of this molecule [55,58].

## 10. Glutathione as a Key Molecule for Redox Status Modulation

GSH is considered a central component and a major orchestrator of cellular redox status. This universal molecule is present in all cells in high concentrations, and its level and the ratio of GSH to GSSG forms are viewed as an integral indicator of oxidative stress, which plays a key role in aging mechanisms. Detailed investigation into GSH metabolism and functions should be the basis for the development of geroprotective strategies.

The intracellular distribution of GSH is dynamic: it is synthesized only in the cytoplasm, and transporter proteins distribute GSH throughout organelles [59]. The outer mitochondrial membrane contains a large number of porins (voltage-dependent anion-selective channels, or VDACs), while the inner membrane contains oxoglutarate and dicarboxylate transporters that transport GSH into mitochondria.

GSH relocalization into the nucleus occurs through nuclear pores—receptor complexes associated with Bcl-2 expression. GSH is also found in the endoplasmic reticulum, where it diffuses into the cytoplasm, being activated by Sec61.

Intracellular/extracellular GSH exchange occurs through the functioning of VDACs involving OATPs (organic anion-transporting polypeptides), CFTR (cystic fibrosis transmembrane regulator), and MRPs (multidrug resistance proteins).

The mitochondrial GSH pool is particularly important, as mitochondria are the main source of ROS in aging. When the level of the mitochondrial GSH pool decreases even slightly, cells become susceptible to oxidative damage, and this may trigger intrinsic mechanisms of apoptosis and necrosis. This level decreases by almost 50% with aging [60].

**Figure 3:**
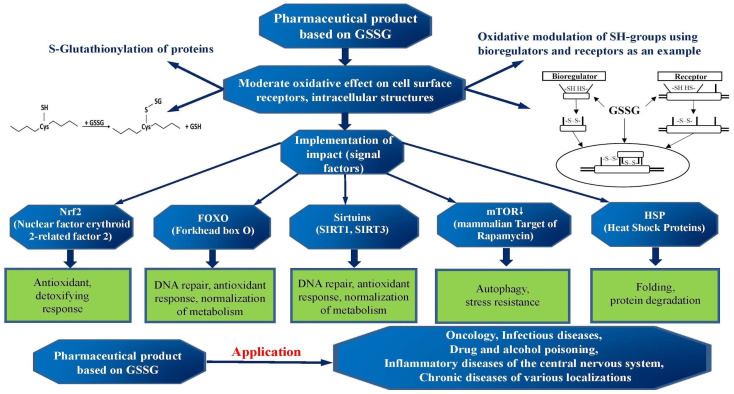
Effects of oxidized glutathione on cellular signaling under moderate oxidative stress. Above are the probable intracellular proteins whose activity level determines the beneficial changes for the cell as a result of moderate oxidative stress under the action of a GSSG-based drug (application fields are given at the bottom of Figure 3).

A key property of GSH is its ability to undergo oxidation and reduction. Three GSH pools are distinguished: GSH, GSSG and conjugates with numerous exogenous and endogenous compounds. And the GSH/GSSG ratio reflects the cellular redox status. A shift toward an increased proportion of GSSG is a key marker of oxidative stress (Figure 3).

The formation of GSSG may be associated with the direct interaction of GSH with ROS. GSH can neutralize hydroxyl radicals (•OH), hydroperoxide radicals (HO_2_•), hydrogen peroxide (H_2_O_2_), nitroxyl radicals (NO•), and peroxynitrite anion (ONOO−) thanks to the presence of “traps” in the form of a thiol group (-SH). Two GSH molecules donate a hydrogen atom, forming a GSSG dimer (2GSH → GSSG + 2H^+^), thereby reducing the ROS molecule. The oxidized form can then be reconverted into two GSH molecules via glutathione reductase and NADPH (GSSG + NADPH + H^+^ → 2GSH + NADP^+^).

Besides its direct interaction with ROS and RNS, GSH is a cofactor for a number of enzymes that enhance its antioxidant properties. One of such enzymes is glutathione peroxidase (GPx), which catalyzes the reduction of H_2_O_2_ and hydroxyperoxides to H_2_O and ROH alcohol. In this reaction, GSH acts as an electron donor.

Glutathione reductase (GR) is an enzyme that catalyzes the reduction of oxidized GSSG to its active form, using NADPH as an H^+^ source. The reaction catalyzed by GR is a source of GSH in the cytosol and some organelles. Glutathione S-transferase (GSTs) catalyzes the conjugation of GSH with a wide range of compounds, which are often formed through cytochrome P450 metabolism.

These reactions detoxify metabolic products, making them water-soluble and more easily excreted. Considerable attention has been paid to glutaredoxins (GRx), small proteins that reduce protein disulfides using GSH as an electron donor.

Glutaredoxin-catalyzed de/glutathionylation is an important event in signal transduction, serving as a primary protective mechanism against the irreversible oxidation of cysteine residues. The age-related decline in the activity of enzymes (a cofactor of which is GSH) further exacerbates redox disorders, impeding GSH restoration from GSSG and cellular antioxidant defense.

GSH depletion is known to be associated with neurodegenerative diseases. Neuroinflammation and neurotoxicity develop when antioxidant defenses are impaired in patients with Parkinson’s disease and amyotrophic lateral sclerosis [61,62,63].

Studies show that geroprotective strategies aimed at modulation of GSH and related enzymes can be highly effective. So, immune system function has been shown to correlate with the state of the GSH system: higher levels of GPx, GR, and GSH, and lower values of GSSG and the GSSG/GSH ratio, improve peripheral blood leukocyte counts in subjects [61].

Table 1 presents the main targets and molecular mechanisms of action of GSSG, as well as their biological effects on the body. These effects include normalizing cellular processes, repairing damaged tissue, and combating age-related diseases. Research suggests that these drugs may be particularly beneficial for various conditions, such as gynecological diseases, inflammatory processes, and cellular aging.

Thus, these findings convincingly demonstrate that GSH is not just a biomarker but also a master regulator of redox status, and its dysfunction underlies many age-associated diseases. This makes the GSH system a key target for geroprotective interventions.

Strategies aimed at maintaining GSH synthesis, restoring the GSH/GSSG ratio, and enhancing the activity of the enzymatic systems it controls appear to be a scientifically sound and promising approach to preventing accelerated age-related organ and tissue involution.

## 11. Glutathione-Based Drugs: A Targeted Geroprotective Effect on Mitochondrial Dysfunction

Two medicines have been added to the Russian State Pharmacopoeia, the active ingredient of which is GSSG and its derivative, and a derivative of GSSG and inosine, inosine-glycyl-cysteinyl-glutamate disodium, which have pronounced antioxidant and immunomodulatory properties.

Inosine and GSSG, as part of inosine-glycyl-cysteinyl-glutamate disodium, are complementary in both pharmacodynamic and pharmacokinetic terms. At the cellular level, the complementarity of GSSG and inosine is expressed in that GSSG increases the affinity of A1, A2A, and A3 receptors for inosine.

A direct or indirect effect of GSSG on inosine-sensitive receptors is manifested in the formation of disulfide cross-links in the structure of the cell surface domain of the receptor protein (inosine-sensitive receptors have at least one disulfide cross-link in their functionally active conformation).

The combined action of these substances at the cellular level translates into positive changes at the tissue or organ level, and throughout the body.

The developed drugs have the ability to maintain cellular redox balance, and they can be viewed as potential geroprotectors for the prevention and treatment of age-associated diseases, as well as for maintaining health throughout life.

Both drugs activate the glutathione antioxidant system, which helps reduce oxidative stress and maintain cellular energy metabolism (GSH, as a major antioxidant, plays a key role in neutralizing free radicals and protecting cells from damage caused by oxidative stress).

Drugs based on GSSG and GSSG derivative and inosine affect the intracellular concentration of calcium ions (Ca^2+^) in macrophages by activating phospholipase A2 and the arachidonic acid cascade. Inhibition of phospholipase A2 with 4-bromophenacyl bromide and glucocorticosteroids such as prednisolone and dexamethasone has been shown to significantly suppress the Ca^2+^ responses induced by these drugs. These findings highlight the role of phospholipase A2 in signaling pathways associated with GSSG-based and GSSG-derivative-and-inosine-based drugs [64,65].

In addition to its antioxidant properties, GSSG-based and inosine-based drugs also exhibit functions of regulating energy metabolism and GABA receptor ligands [66]. This promotes the restoration of cognitive functions and reduces the effects of toxic and hypoxic damage to the central nervous system.

Studies in patients with acute conditions such as toxic coma and pancreatitis demonstrated the ability of GSSG-based and GSSG-derivative-inosine-based drugs to accelerate recovery and improve metabolic parameters, which may serve as an indirect indication of their potential for maintaining optimal quality of life during aging.

Haloperidol (a Sigma-1 receptor antagonist) has also been found to suppress the Ca^2+^ response induced by a derivative drug based on GSSG and inosine in macrophages, which points to the involvement of sigma-1 receptors in its action [67]. The GSSG-based drug in the study demonstrated the ability to prevent pathological changes caused by toxic effects of cytostatics, including developmental, cognitive, and adaptive disorders in animal offspring.

These findings point to the important role of the GSSG-based drug, and especially the GSSG derivative and inosine, in protecting cells from oxidative and mitochondrial stress, which is critical for maintaining mitochondrial function [72]. A drug based on GSSG and inosine demonstrated obvious hepatoprotective properties in patients with toxic liver injury, which is associated with maintaining mitochondrial metabolism and reducing cellular damage [73].

The GSSG-based drug shows potential for molecular prevention of inflammaging, contributing to the correction of immune disorders and reducing the severity of chronic inflammation. It also improves the effectiveness of antibacterial therapy and promotes the elimination of pathogens, which contributes to a decrease in chronic inflammation levels [74].

In studies using skin disease (psoriasis) models, a GSSG-based drug corrected the expression of cell cycle proteins associated with inflammation and proliferation, thereby reflecting its role in regulating inflammatory processes [68].

It has been shown that the age-related decrease in GSH levels and imbalance in its system play an important role in the development of oxidative stress leading to pathological aging.

Thus, a targeted impact on the GSH system is one of the most substantiated approaches to geroprotection. Pharmacological solutions based on GSSG, GSSG derivatives, and inosine, as GSSG pharmaceutical forms, are promising agents for correcting redox status by a targeted impact on the GSH system.

Unlike direct administration of GSH, which is poorly absorbed, GSSG serves as a substrate for reduction to active GSH by the GR enzyme, effectively increasing the intracellular GSH concentration. Moreover, GSSG administration becomes a natural signal of oxidative stress for the cell and can indirectly activate the NRF2 pathway.

Activation of the NRF2 pathway, as described above, stimulates not only the cellular antioxidant defense via GSH but also recruits SOD enzymes, catalase, and heme oxygenase-1. It is important to note that unlike direct and persistent activation of NRF2, which may have negative consequences, GSSG administration seems to mimic natural physiological signals, resulting in temporary and balanced activation of this pathway.

Thus, drugs based on GSSG and GSSG derivative and inosine not only replenish the GSH pool but also stimulate the production of numerous enzymes involved in combating cellular oxidative stress. For instance, toxic liver damage resulting from the metabolism of anti-tuberculosis drugs and accompanied by ROS production was reduced by the administration of these drugs [75]. In patients with fatty liver disease combined with liver fibrosis, the administration of a drug based on GSSG and inosine significantly reduced the disease severity, exerting an anti-inflammatory and antifibrotic effect [76].

Liver damage resulting from ethanol detoxification was also corrected by a drug based on GSSG derivative and inosine. It significantly reduced the activity of alanine aminotransferase, aspartate aminotransferase, alkaline phosphatase, gamma-glutamyl transpeptidase, and direct bilirubin three hours after administration. The same study showed improved metabolism, with a decrease in lactate levels and an increase in potassium content in the blood electrolyte profile [77].

Correction of toxic liver damage demonstrates the ability of these drugs to protect organs from injuries, the mechanisms of which are similar to the processes of accelerated organ involution with aging. It was also shown that administration of a GSSG-based drug altered GR activity in influenza: after treatment, it decreased but remained higher than in the group of subjects receiving only antiviral therapy [78].

This may be evidence of an increase in the activity of GSH system enzymes and a potential enhancement of cellular antioxidant defense.

Chronic alcohol intoxication is associated with changes in the functional and metabolic activity of peripheral blood neutrophils, which are typical of an inflammatory response and characterized by increased oxygen-dependent activity and decreased cell phagocytic activity. The use of a GSSG-based drug as an antioxidant resulted in normalization of granulocyte functional activity parameters in an animal model of combined experimental acute pancreatitis and chronic alcohol intoxication [79].

The experiment provided important evidence of a geroprotective targeted effect of drugs based on GSSG and GSSG derivative and inosine through normalization of mitochondrial protein expression. Administration of the drugs to older rats resulted in increased expression of proteins—TOM 20 and 70, VDAC, DRP1, Parkin, PINK1, and prohibitin—which are major regulators of mitochondrial function (mitochondrial fission, mitochondrial DNA stability, and protection against alterations in the internal potential of the mitochondrial membrane) [69,70].

A study of the effect of these two drugs on the expression of key signaling molecules involved in inflammaging mechanisms showed that the primary target of the geroprotective action of the drug based on the GSSG derivative and inosine is prohibitin, while the targets of the geroprotective action of the GSSG-based drug are hTERT and prohibitin. The administration of the drugs into the endometrial cell culture under conditions of inflammaging modeling resulted in a return of the expression values of hTERT and prohibitin markers to the values of the control group, which pointed to the activation of repair processes and cells’ fight against oxidative stress (Table 2) [71].

Further comprehensive studies of drugs based on GSSG and its derivatives will help clarify the mechanism of their effect on the expression of key signaling gerotropic molecules and, thereby, expand indications for their use as therapeutic agents for the prevention of premature aging and for the treatment of age-associated diseases.

These clinical and experimental data demonstrate that correction of the GSH system with GSSG-based drugs is effective for a wide range of pathologies associated with oxidative stress, from toxic liver damage to viral infections. Their ability to restore redox balance and cellular function confirms the high potential of drugs based on GSSG and GSSG derivative and inosine as proven geroprotective agents.

## 12. Conclusions

Numerous studies confirm that mitochondrial dysfunction and associated redox status imbalance are major pathogenetic factors in the aging process. Disruption of mitochondrial quality control, defects in mitophagy, and the accumulation of dysfunctional organelles initiate oxidative stress, which then leads to macromolecular damage and chronic systemic inflammation (inflammaging), which creates the foundation for the development of a wide range of age-associated diseases.

In this context, strategies aimed at modulating redox homeostasis appear to be a very promising approach to the development of geroprotectors.

The glutathione system plays a pivotal role in maintaining cellular redox status, where the GSH/GSSG ratio is an indicator of oxidative stress. The age-dependent decline in the mitochondrial GSH pool and the activity of associated enzymes (glutathione peroxidase, glutathione reductase, and glutathione-S-transferase) critically impairs antioxidant defenses, increasing cellular vulnerability to apoptosis and necrosis.

An overview of various classes of geroprotective compounds, including flavonoids, NRF2 pathway activators (KEAP1-NRF2), NAD^+^ level and sirtuin modulators, and mTOR inhibitors (rapamycin), has shown their significant potential in experimental models. However, their clinical application is limited by pharmacokinetics, bioavailability, and a lack of large-scale randomized trials.

In this context, drugs based on GSSG and GSSG derivative and inosine are of particular interest. Unlike exogenous GSH, GSSG is effectively absorbed by cells, acting as a substrate for glutathione reductase, and can indirectly activate the NRF2 pathway, potentiating the expression of a wide range of antioxidant and detoxifying genes. Experimental and clinical data demonstrate their efficiency in correcting pathologies associated with oxidative stress and mitochondrial dysfunction, including toxic liver damage, neurodegenerative processes, and immune disorders, which becomes evident in normalization of biochemical markers, improved mitochondrial biogenesis, and enhanced activity of glutathione system enzymes.

Aging is increasingly recognized as a complex, regulated process rather than a simple accumulation of molecular damage. One of the most influential systemic approaches to aging was proposed by Vladimir M. Dilman [92], who viewed aging as a consequence of progressive dysregulation of homeostatic control systems. Dilman’s theory described aging as a consequence of impaired feedback sensitivity and persistent hyperfunction within neuroendocrine regulatory systems. Current evidence from redox biology provides a molecular basis for this concept, identifying hypothalamic redox dysregulation as a central initiating event in age-related homeostatic imbalance [93]. Physiological hypothalamic function relies on tightly controlled, temporally structured redox signaling. Reactive oxygen species act as second messengers linking nutrient availability, hormonal cues, and neuronal activity. With aging, this regulation deteriorates: antioxidant enzyme rhythms in the suprachiasmatic nucleus become desynchronized, indicating loss of circadian control over redox signaling at the level of the central clock [94,95].

Within the framework of the redox code, such changes reflect reduced fidelity of redox information processing, whereby adaptive, pulsatile signaling shifts toward sustained oxidative pressure. This loss of redox precision promotes persistent activation of metabolic and neuroendocrine pathways, consistent with impaired feedback inhibition [1]. Metabolic stressors further reinforce this process. Overnutrition and age-related iron accumulation in arcuate nucleus neurons increase mitochondrial ROS production, triggering redox-dependent transcriptional reprogramming (e.g., FoxO1 activation and AgRP upregulation) [96] and resetting hypothalamic metabolic set points. These changes contribute to insulin and leptin resistance, reduced energy expenditure, and chronic activation of the hypothalamic–pituitary–adrenal axis [97].

Collectively, these findings support a model in which age-associated disruption of hypothalamic redox homeodynamics drives central regulatory hyperfunction, as originally proposed by Dilman. Thus, his systemic aging theory is increasingly substantiated by contemporary data demonstrating that loss of redox and circadian precision within the hypothalamus initiates endocrine, metabolic, and immune dysregulation.

Thus, the combination of V.M. Dilman’s ideas and modern concepts of redox status can be considered as a holistic model of aging as a process of loss of regulatory sensitivity and homeodynamic balance.

Within this system, GSSG mimetics act as core molecules: they simultaneously correct fundamental mechanisms of aging and enhance the action of existing drugs. This makes them a primary geroprotective tool for the prevention and treatment of age-associated diseases.

Undoubtedly, when discussing the broad prospects of studying various natural factors and pharmacological agents as effective geroprotective agents, it is necessary to mention the concept of hormesis and the role of mitochondria in the implementation of this biological phenomenon.

Mitochondrial dysfunction in aging is also linked to hormesis, the concept that the use of low-toxicity drugs triggers beneficial compensatory responses that not only repair the initiating damage but also make the cell more resistant to damage. In recent years, hormesis has received growing attention as a central concept in aging and translational medicine, describing how low or mild stressors can activate adaptive cellular responses that enhance resilience, whereas excessive stress is detrimental [98].

As is well known, hormesis (from the Greek ορμεσις—rapid movement, striving) refers to a biological phenomenon characterized by a two-phase response of a living system to an environmental stress factor (or factors), where low doses of the factor have a beneficial effect, while high doses lead to negative manifestations. Hormesis has been observed in various groups of organisms, at virtually all levels of life organization: from subcellular structures to ecosystems, demonstrating its evolutionary relevance [99,100].

Nutritional hormetins—bioactive dietary compounds that exploit this principle—induce beneficial adaptive responses, and when specifically targeting mitochondria, they are referred to as mitohormetins, activating mitochondrial pathways to improve cellular and systemic health. Mild or transient mitochondrial stress is increasingly recognized as a physiological signal that triggers conserved adaptive programs, including the mitochondrial unfolded protein response (UPRmt) and the mitochondrial arm of the integrated stress response (ISRmt), which together coordinate mitochondrial proteostasis, bioenergetic function, and nuclear transcriptional reprogramming [101].

Through this mechanism, nutritional antioxidants such as polyphenols (resveratrol, quercetin, catechins), sulforaphane, melatonin, omega-3 fatty acids, capsaicin, and probiotic- or fermentation-derived metabolites activate compensatory stress-response pathways that enhance mitochondrial efficiency, antioxidant defenses, and metabolic flexibility. Central to these adaptive responses is the redox code governed by the glutathione redox couple (GSH/GSSG), which integrates mitochondrial, cytosolic, and systemic redox states and translates redox fluctuations into context-dependent signaling outputs. Fine-tuning of the GSH/GSSG ratio enables redox-sensitive activation of Nrf2 and coordinated transcriptional programs regulating antioxidant enzymes, glutathione metabolism, mitochondrial quality control, and immune homeostasis. UPRmt- and ISRmt-driven signaling promotes the upregulation of mitochondrial antioxidants, enzymes involved in glutathione synthesis and recycling, and metabolically tailored anti-inflammatory pathways, facilitating stress resolution while preventing chronic immune activation. Nutrient-induced mitohormesis preserves systemic redox balance, restrains chronic low-grade inflammation (inflammaging), and enhances interorgan communication through mitokines, mitochondrial-derived peptides, extracellular vesicles, and neuroendocrine factors such as FGF21, GDF15 and MOTS-c, coordinating metabolic adaptation and tissue resilience throughout the body [102]. Building on these systemic hormetic signals, certain bioactive dietary compounds can target the nervous system, activating adaptive neuronal stress responses that improve brain resilience. These compounds, referred to as neuronutrients, modulate neurohormesis—defined as the process by which mild, adaptive neuronal stress enhances cellular defense, antioxidant responses, and metabolic flexibility—thereby protecting neurons from oxidative stress, neuroinflammation, and blood–brain barrier dysfunction. Dose-dependent flavonoids and polyphenols upregulate phase II antioxidant genes (e.g., HO-1) and sirtuin-1 (Sirt1), promoting neuronal resilience and adaptive stress responses [103]. Neuronutrition, as a personalized nutritional approach, leverages these mechanisms to prevent or delay neurodegenerative and neuropsychiatric disorders, including Alzheimer’s disease, Parkinson’s disease, and autism spectrum disorders, by enhancing brain redox resilience and cognitive function [104].

Collectively, nutritional antioxidants and neuronutrients acting as hormetic agents harness mitohormesis, the GSH/GSSG redox code, interorgan signaling, and inflammaging control to reinforce endogenous defense systems, improve brain and systemic resilience, support cognitive and metabolic health, and promote healthy aging, highlighting the translational potential of personalized nutritional strategies for neuroprotection and longevity.

Therefore, the targeted modulation of the GSH system using drugs based on GSSG and GSSG derivative and inosine can be viewed as a valid pathogenetic strategy for targeted geroprotection aimed at slowing cellular senescence and maintaining the functional homeostasis of organs and tissues.

Prospects for further research include an in-depth investigation of molecular mechanisms of glutathione derivative action and controlled clinical trials to evaluate their effectiveness directly in the context of prolonging human life expectancy and quality of life.

## Figures and Tables

**Table 1:** Molecular/cellular targets and biological effects of glutathione.

Targets	Molecular Mechanism	Biological Effects
A1, A2A, and A3 receptors for inosine	Increased affinity for inosine	Improving tissue and organ functions [64,65]
GSSG	Binds to electrophilic compounds (drugs, toxins, oxidation products) via GST enzymes, forming water-soluble conjugates that are removed from the cell. This reduces the toxicity of xenobiotics and protects cellular structures	Reduction in oxidative stress and stabilization of the redox state [65]
Calcium ions	Activates phospholipase A2	Alteration of macrophage calcium responses [64,65]
GABA-receptors	Acts as a ligand for GABA receptors	Improved cognitive function and reduced CNS damage [66]
Sigma-1 receptors	Modulation of calcium response	Protection against oxidative stress [67]
Cell cycle proteins	Effects on signaling pathways associated with proliferation, apoptosis, differentiation and immune response, including MAPK, PI3K/AKT, TOR and HIF-1	Regulation of inflammatory processes [68]
Mitochondrial proteins	Normalization of expression of TOM 20, TOM 70, VDAC, DRP1, Parkin, PINK1 and prohibitin) [69,70]	Maintaining mitochondrial function, including division and stability. Prevention of cytotoxic damage
Telomerase reverse transcriptase (hTERT)	Restoration of activity to control levels [71]	Promotes cell proliferation and tissue repair

**Table 2:** Therapeutic action of glutathione-based drugs.

Potential Therapeutic Agents	Biological Effects	Literary References
Dihydroquercetin	Activation of the antioxidant system, inhibition of pro-inflammatory and pro-apoptotic pathways, reduction in mitochondrial ROS production, activation of mitophagy.	[80,81]
Rapamycin	An inhibitor of the mTOR target of rapamycin significantly increases lifespan.	[82]
Resveratrol	Resveratrol activates the sirtuin family, which improves mitochondrial function, induces mitophagy, reduces oxidative stress, and suppresses inflammatory pathways, including caspase-3 and NF-κB. It also has neuroprotective properties.	[83,84]
Vitamin A	It suppresses ferroptosis and provides neuroprotection; vitamin A metabolites affect the cell cycle of skin cells, wound healing and stem cells.	[85,86,87]
Vitamin C	Reduction in oxidative stress, activation of transcription factors (Nrf2, Ref-1), support of the activity of other antioxidants.	[88]
Vitamin E	Protection against membrane peroxidation.	[89]
Melatonin	Antioxidant and anti-inflammatory effects, neuroprotection, suppression of inflammaging and prevention of insulin resistance.	[90]
Precursors of NAD^+^	Antioxidant and anti-inflammatory effects, neuroprotection, suppression of inflammaging and prevention of insulin resistance	[91]
Glutathione-based preparations	Restoration of redox balance, mitochondrial protection, immunomodulation, anti-inflammatory effect	[69]

## Data Availability

No new data were created or analyzed in this study. Data sharing is not applicable to this article.
